# Relationships between kinetic constants and the amino acid composition of enzymes from the yeast *Saccharomyces cerevisiae* glycolysis pathway

**DOI:** 10.1186/1687-4153-2012-11

**Published:** 2012-08-06

**Authors:** Peteris Zikmanis, Inara Kampenusa

**Affiliations:** 1 Institute of Microbiology and Biotechnology, University of Latvia, Kronvalda Boulevard 4, Riga, LV-1010, Latvia

**Keywords:** Michaelis-Menten constant, Turnover number, Specificity constant, Glycolytic enzymes, Sequence-dependent properties, Multivariate relationships

## Abstract

The kinetic models of metabolic pathways represent a system of biochemical reactions in terms of metabolic fluxes and enzyme kinetics. Therefore, the apparent differences of metabolic fluxes might reflect distinctive kinetic characteristics, as well as sequence-dependent properties of the employed enzymes. This study aims to examine possible linkages between kinetic constants and the amino acid (AA) composition (AAC) for enzymes from the yeast *Saccharomyces cerevisiae* glycolytic pathway. The values of Michaelis-Menten constant (*K*M), turnover number (*k*cat), and specificity constant (*k*sp = *k*cat/*K*M) were taken from BRENDA (15, 17, and 16 values, respectively) and protein sequences of nine enzymes (HXK, GADH, PGK, PGM, ENO, PK, PDC, TIM, and PYC) from UniProtKB. The AAC and sequence properties were computed by ExPASy/ProtParam tool and data processed by conventional methods of multivariate statistics. Multiple linear regressions were found between the log-values of *k*cat (3 models, 85.74% < *R*adj.2 <94.11%, *p* < 0.00001), *K*M (1 model, *R*adj.2 = 96.70%, *p* < 0.00001), *k*sp (3 models, 96.15% < *R*adj.2 < 96.50%, *p <* 0.00001), and the sets of AA frequencies (four to six for each model) selected from enzyme sequences while assessing the potential multicollinearity between variables. It was also found that the selection of independent variables in multiple regression models may reflect certain advantages for definite AA physicochemical and structural propensities, which could affect the properties of sequences. The results support the view on the actual interdependence of catalytic, binding, and structural residues to ensure the efficiency of biocatalysts, since the kinetic constants of the yeast enzymes appear as closely related to the overall AAC of sequences.

## Introduction

According to the concepts of systems biology, metabolic fluxes are net sums of underlying enzymatic reaction rates represented by integral outputs of three biological quantities which interact at the level of enzyme kinetics: kinetic parameters, enzyme and reactant concentrations [1]. Integrated view of enzymes suggests to consider them as dynamic assemblies whose variable structures are closely related to catalytic functions [2,3]. It is therefore an important task to extend the knowledge of the enzyme sequence, structure and function relationships which allow to specify a chemical mechanism of catalytic reaction and to be predictive for targeted modification of enzymes [4]. Site-directed mutagenesis has proved to be a powerful tool to probe certain amino acids (AA) within an enzyme, yet still somewhat less focusing on other residues and, therefore, tempted to ignore the actual interdependence of catalytic, binding, and structural residues being considered as a key feature of such complex cooperative systems [2,3,5]. Moreover, statistical evaluation of the relation between functionally and structurally important AA of the enzyme sequences reveals contribution of the catalytic residues to the structural stabilization of the respective proteins, which indicates both residue sets as rather overlapping than segregated [6]. In addition, the modest success of creating artificial enzymes also points to currently unknown, probably crucial, parameters that could significantly affect enzyme catalysis [7]. AA composition (AAC) is a simplest attribute of proteins among the so-called global sequence descriptors [8] which represents the frequencies of occurrence of the natural AA thereby creating a 20-dimensional feature for a given protein sequence [8,9]. AAC appears as a simple, yet powerful feature for a successful prediction of several protein properties, including protein folding and mutual interactions [10-12].

On the other hand, these complex events can be measured in many respects, including protein conformational heterogeneity and structural dynamics [7,13,14]. For these reasons, there could be certain links between the enzyme kinetic constants and AAC of the sequences. The goal of this study was to check this assumption.

## Methods

The dataset consisted of the enzyme characteristics, representing the yeast *Saccharomyces cerevisiae* glycolysis pathway, together with the reaction directly branching (pyruvate carboxylase) from it. It includes the data for the following enzymes: Hexokinase (HXK, EC 2.7.1.1), Glyceraldehyde-3-phosphate dehydrogenase (GADH, EC 1.2.1.12), 3-phosphoglycerate kinase (PKG, EC 2.7.2.3), Phosphoglycerate mutase (PGM, EC 5.4.2.1), Enolase (ENO, EC 4.2.1.11), Pyruvate kinase (PK, EC 2.7.1.40), Pyruvate decarboxyase (PDC, EC 4.1.1.1), Triose-phosphate isomerase (TIM, EC 5.3.1.1), and Pyruvate carboxylase (PYC, EC 6.4.1.1). The kinetic constants and the enzyme AA sequences were taken from the BRENDA [15] and UniProtKB [16] databases, respectively. The numerical values of kinetic constants retrieved from BRENDA and the UniProtKB accession numbers of enzyme sequences are summarized in Additional file 1: Table S1. The relatively limited volume of this dataset is due to the fact that only these glycolytic enzymes from *S. cerevisiae* are currently represented in BRENDA database [15] by both fundamental constants [17]: the turnover number (*k*cat), the Michaelis-Menten constant (*K*M) and, consequently, the derived specificity constant (*k*sp = *k*cat/*K*M) [17,18]. The values of *k*cat and *K*M obtained from the same literature source were used for the direct calculation of *k*sp. If the several kinetic constants with the different numerical values come from various literature sources (m*n) values for *k*sp were calculated, where *m* and *n* represent the numbers of *k*cat and *K*M, respectively (Additional file 1: Table S1). In this way, the calculated smallest and largest *k*sp values were excluded from subsequent use to form a more even balance for the number of sequences under study. Consequently, 16 *k*sp values were included in the data set (Additional file 1: Table S1).

The AAC (frequencies of AA occurrence) of sequences was computed using ExPASy/ProtParam tool [19]. The average AA property, *P*ave(*i*), for each sequence (or an extracted group of AA) was computed using the standard formula [20], where *P*(*j*) is the property value for *j*th residue and the summation over *N*, the total number of residues in a protein.

The data were processed by correlation analysis (parametric and non-parametric) using the Statgraphics®Plus (Manugistics Inc., Maryland, USA) and SPSS 11.0 for Windows (SPSS Inc., Illinois, USA) and subjected to the multiple linear regression analysis using the same software. Explanatory variables in the models were selected by stepwise forward selection procedures by finding the significant one-variable models (20 AA × 3 kinetic constants) as well as significant two-variable models (190 possible ways/*C*(20,2)/to arrange 20 AA in groups of 2 at a time for each kinetic constant). The best three-variable models were formed by adding another variable one-by-one from the remaining ones and the variables that yield the greatest increase in the adjusted *R*2 value were included. And so forth to obtain the four-variable and larger models until no variables could increase the criterion. The logarithmic transformation of the kinetic constant values was used to increase the normality of the dependent variables. The Fisher’s *F*-test for analysis of variance (ANOVA) was performed to evaluate the statistical significance of regression models and the Student’s *t*-test was employed to check the significance of regression coefficients. The leave-one-out cross-validation (LOOCV) procedure was employed to validate developed regression models [21]. The linear plots of the actual kinetic constants against those predicted by the multiple regression models were used throughout the study to assess the goodness-of-fit for observed multivariate relationships according to adjusted *R*2 values. Conventional non-parametric tests, including the Friedman ANOVA for ranks and the Wilcoxon signed rank test, were used to evaluate the *P*ave(*i*) for each protein in respect of the AA groups selected/non-selected as the predictor variables.

The *p* values < 0.05 were considered to be statistically significant for both parametric and non-parametric tests.

A conventional single letter code was used throughout to denote AA representing their frequencies of occurrence as the independent variables.

## Results

**Figure 1:**
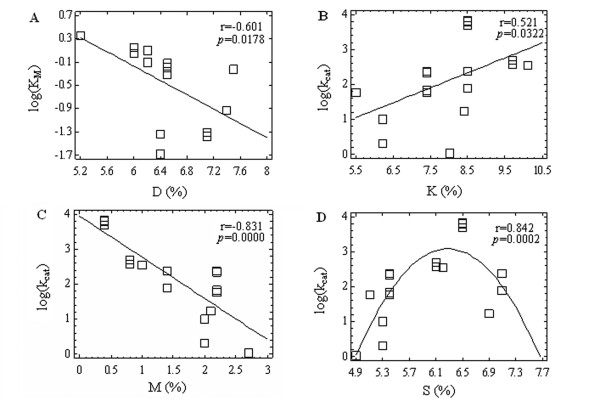
**The relationships between kinetic constants and the frequencies of individual AA.** Bivariate correlations between the log-values of kinetic constants and frequencies of occurrence for individual AA in the yeast *S. cerevisiae* enzyme sequences, where *K*M is the Michaelis-Menten constant **(A)** and *k*cat is the catalytic constant **(B–D)**. All the linear correlations are significant at the non-parametric assessment (Kendall’s τ, Spearman’s ρ correlation coefficients).

Already a bivariate correlation analysis of 60 possible relationships (3 kinetic constants × 20 AA) revealed 12 significant parametric and/or rank correlations, confirming that the enzyme constants can be linked up even with the individual AA frequencies. Furthermore, the observed relationships (Figure 1) for different AA can be as direct (B) as well the reverse (A, C) or even a non-linear (D).

**Figure 2:**
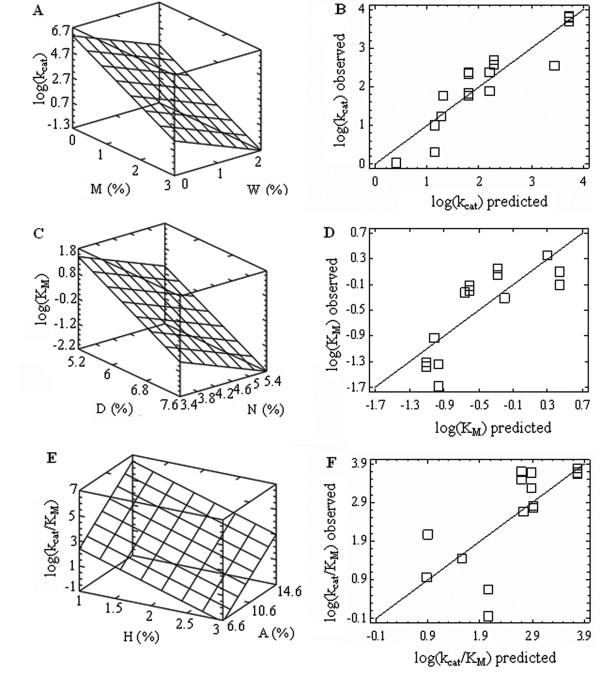
**The relationships between kinetic constants and frequencies of two AA.** The multiple linear regressions showing changes of the log-values of kinetic constants as dependent variables upon the frequencies of occurrence for two AA in the yeast *S. cerevisiae* sequences, where *k*cat is the catalytic constant **(A)**, *K*M is the Michaelis-Menten constant **(C)**, and *k*sp = *k*cat/*K*M is the specificity constant **(E)**. The observed versus predicted plots **(B,D,F)** for the values of dependent variables (*k*cat, *K*M, and *k*sp, respectively). The predicted values were calculated from the regression equations: log(*k*cat) = 5.556 –1.620*M −0.984*W (*R*adj.2 = 82.88%, *p* = 0.0000); log(*K*M) = 8.593 –0.596*N −0.998*D (*R*adj.2 = 53.72%, *p* = 0.0039); log(*k*cat/*K*M) = 0.818 +0.501*A −1.736*H (*R*adj.2 = 46.50%, *p* = 0.0068). All the multiple and pair correlations (A–F) are significant at the non-parametric assessment (Kendall's τ, Spearman's ρ correlation coefficients).

**Figure 3:**
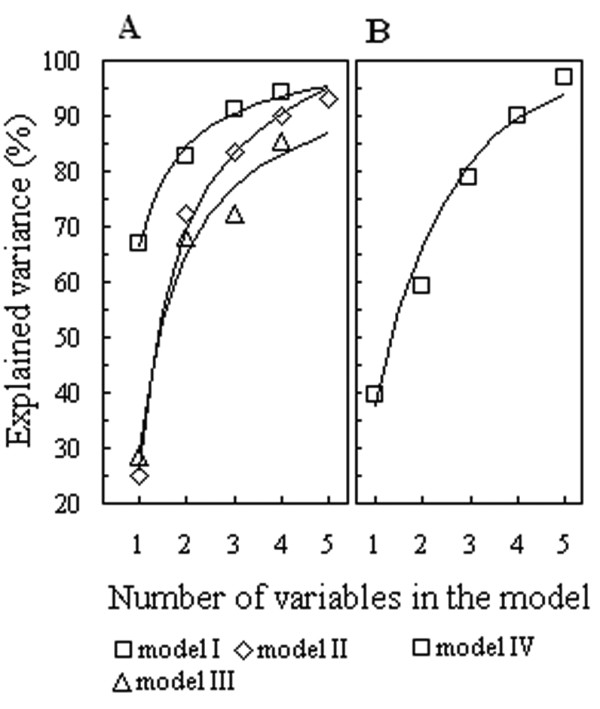
**The changes of explained variance upon the growing number of variables in the models.** Relationships between an increase in the percentage of explained variance and the number of independent variables (AA frequencies of occurrence) included in multiple regressions, where **A** and **B** represent the variety of cases for log(*k*cat) and log(*K*M), respectively. Variables in the models: model I: 1 – M, 2 – M, W, 3 – M, W, R, and 4 – M, W, R, L; model II: 1 – T, 2 – T, V, 3 – T, V, H, 4 – T, V, H, A, and 5 – T, V, H, A, K; model III: 1 – H, 2 – H, A, 3 – H, A, E, and, 4 – H, A, E, V; model IV: 1 – D, 2 – D, N, 3 – D, N, W, 4 – D, N, W, L, and 5 – D, N, W, L, A.

**Figure 4:**
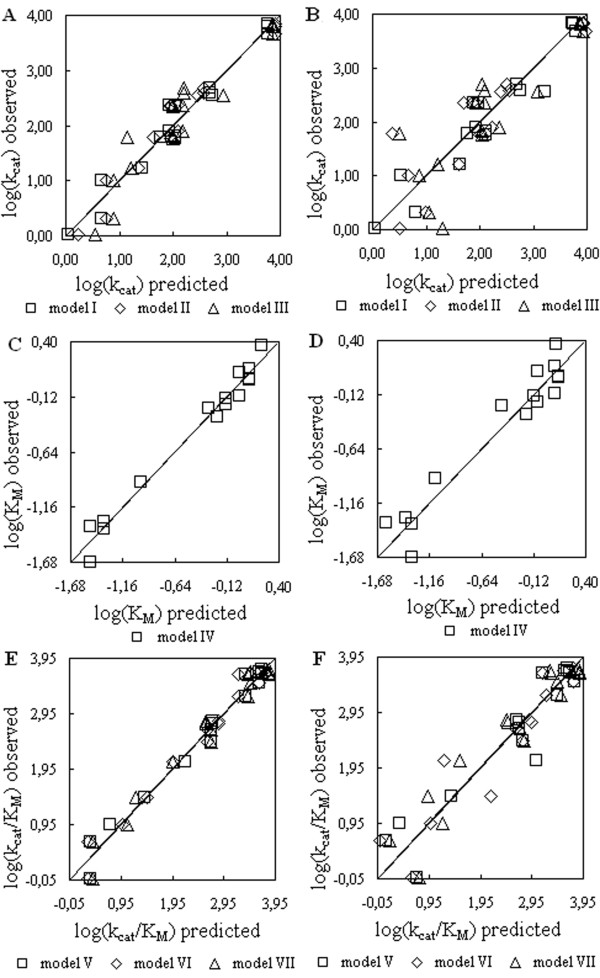
**Linear plots of the actual kinetic constants against those predicted by linear regression models.** The observed versus predicted plots (**A,C,E**) for the values of dependent variables log(*k*cat), log(*K*M), and log(*k*cat/*K*M), respectively. The predicted values were calculated from the statistically robust model equations as specified in Table 1, including those obtained by the LOOCV of models (**B,D,F**).

Subsequent analysis of the data by means of the forward selection procedures showed that the stepwise inclusion of additional variables leads to a statistically significant multiple regression, where the kinetic constants appear to depend on two or more AA frequencies, thus substantially increasing the proportion of the “explained” variance (Figures 2 and 3). Furthermore, the increasing adjusted *R*2 values indicate that the “explained” variance substantially rises with the growing number of variables in the regression model, although in a nonlinear proportion, due to a more pronounced contribution of the few “strongest” AA frequencies (Figure 3). Therefore, four to six variables turned out to be enough to form statistically robust multiple linear regression models linking the enzyme kinetic constants with the AAC of corresponding sequences (Table 1). The matching quality of the data obtained by the proposed models was evaluated by the linear plots (Figure 4,A,C,E) of the actual kinetic constants against those predicted by proposed regression models (Table 1). The highly significant adjusted *R*2 values also point out that the models (Table 1) adequately represent the actual relationships between the AAC and kinetic constants of the enzymes, since only a relatively small proportion (3.30–14.26%) of the total variance remains unexplained. In addition, the validation of models using the LOOCV procedure although resulted in the certain reduction of the *R*2 values (Table 1, Figure 4B,D,F), but still remained within the limits of high (*p* < 0.00001) statistical significance.

**Table 1:** The characteristics of the obtained models

**Regression model**	**Dependent variable**	**Parameters**^**a**^	**Regression coefficient**	**S.E.**	***t*value**	**P value**	**R**^**2**^**%**	**R**_**adjusted**_^**2**^**%**	**VIF**^**b**^	**R**^**2**^**%**^**c**^	**R**_**adjusted**_^**2**^**%**^**c**^
I	log(k_cat_)	*constant*	5.2073	0.5003	10.408	0.0000	95.58	94.11		90.72	90.10
		M	−1.6219	0.1169	−13.879	0.0000			1.853		
		W	−0.5258	0.2147	−2.449	0.0307			3.329		
		R	0.3558	0.07329	4.855	0.0004			1.103		
		L	−0.1697	0.06309	−2.691	0.0196			2.180		
II	log(k_cat_)	*constant*	3.9385	1.3200	2.984	0.0124	95.22	93.05		80.32	79.01
		T	−0.4482	0.07274	−6.161	0.0001			2.851		
		V	0.2756	0.05350	5.151	0.0003			1.530		
		H	−1.3861	0.2088	−6.639	0.0000			2.003		
		A	0.2840	0.06859	4.141	0.0016			1.868		
		K	−0.2333	0.09633	−2.422	0.0339			2.857		
III	log(k_cat_)	*constant*	−6.3103	1.7275	−3.653	0.0033	89.30	85.74		71.62	69.73
		A	0.4367	0.07955	5.489	0.0001			1.224		
		H	−0.9759	0.3015	−3.237	0.0071			2.034		
		V	0.2728	0.07752	3.519	0.0042			1.564		
		E	0.5900	0.1564	3.773	0.0027			1.498		
IV	log(K_M_)	*constant*	13.2588	0.8236	16.098	0.0000	97.88	96.70		93.18	92.66
		D	−1.1379	0.06612	−17.209	0.0000			1.365		
		N	−0.9961	0.07256	−13.729	0.0000			1.932		
		W	1.0535	0.08387	12.561	0.0000			1.948		
		L	−0.2347	0.03077	−7.628	0.0002			2.140		
		A	−0.09888	0.02288	−4.321	0.0019			1.093		
V	log(k_cat_/K_M_)	*constant*	−11.0119	1.5657	−7.052	0.0001	97.77	96.29		88.86	88.06
		A	−0.5525	0.05736	9.632	0.0000			1.705		
		H	−1.2042	0.1817	−6.626	0.0001			2.082		
		R	1.1894	0.1006	11.829	0.0000			2.373		
		G	0.6911	0.09445	7.317	0.0000			2.520		
		Q	−0.5142	0.1009	−5.098	0.0006			1.672		
		N	0.4252	0.1246	3.412	0.0077			2.176		
VI	log(k_cat_/K_M_)	*constant*	9.4887	0.8188	11.589	0.0000	97.69	96.15		88.86	88.07
		L	−0.4399	0.05548	−7.929	0.0000			1.902		
		T	−0.9367	0.07023	−13.338	0.0000			3.267		
		N	1.1552	0.1032	11.194	0.0000			1.437		
		W	−1.0394	0.2182	−5.012	0.0007			3.420		
		Q	−0.3207	0.1191	−2.692	0.0247			2.244		
		F	−0.2690	0.09349	−2.877	0.0183			1.349		
VII	log(k_cat_/K_M_)	*constant*	2.5597	0.8288	3.088	0.0115	97.00	96.50		90.44	89.77
		T	−0.8156	0.06297	−12.953	0.0000			2.249		
		Q	−0.7700	0.1050	−7.331	0.0000			1.495		
		C	2.4452	0.2845	8.593	0.0000			3.581		
		N	0.5745	0.1162	4.943	0.0006			1.561		
		A	0.2605	0.06600	3.946	0.0027			2.027		

Elements and the statistical indices for multiple linear regression models which link the log-values of kinetic constants and the AAC of the yeast *S. cerevisiae* enzyme sequences.

a Elements of multiple linear regression which represent the frequencies of AA (a single letter code) occurrence in the yeast *S. cerevisiae* enzyme sequences and the constant (intercept) of equation.

b The variance inflation factor which indicates the impact of multicollinearity between the independent variables [22].

c Obtained by the LOOCV [21] of models.

It is noted that rather small or moderate values of the variance inflation factor (VIF) [22] (Table 1) also indicate that the observed multivariate relationships are not significantly affected by the multicollinearity of independent variables.

The ANOVA for the regression models are summarized in Additional file 2: Table S2.

**Figure 5:**
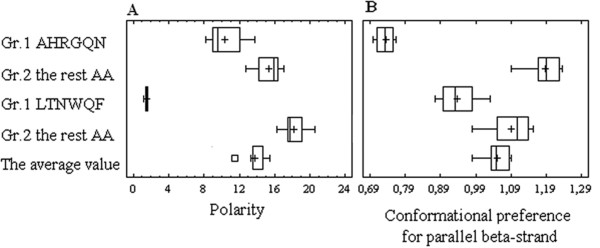
**Different contributions of the selected and non-selected AA into the properties of enzyme sequences.** The Box-and-Whisker plot of the average AA property estimates for the selected/non-selected groups of independent variables in respect of the *k*cat/*K*M regression models (models V and VI, Table 1). The upper and lower bounds of the bars represent maximum and minimum values of estimates, the upper and lower bounds of each box represent the upper and lower quartiles of estimations and the lines in the middle of each box represent the median values. The effects of group selection and all pair differences between the groups are significant (Friedman ANOVA and Wilcoxon signed rank tests, respectively).

Comparison of multiple regression models (Table 1) showed that they include a broad, although uneven, representation of AA where some of them occur more frequently, while others rarely or not, thus creating ranked series (A > N > Q, H, L, T, W > R, V > D, C, E, G, K, M, F > I, P, S, Y) under the downward distribution of AA occurrences. Moreover, it was found that ranked differences of AAC are reflected in their rankings for physicochemical and structural propensities as confirmed by significant multiple rank as well as by parametric correlations: Kendall’s τ1.23 = 0.372 (*p <* 0.05), Spearman’s ρ1.23 = 0.609 (*p <* 0.01), Pearson’s *r*1.23 = 0.623 (*p <* 0.01), where 1 is the AA occurrence, 2 is the average flexibility index [23], and 3 is the propensity for AA hydrophobicity (OMH) [24]. These correlations indicate that the selection of independent variables in multiple regression models may reflect certain advantages for definite AA properties, which, in turn, could affect the overall properties of sequences. This possibility was also confirmed by assessing the enzyme sequences as well as the groups of the selected and non-selected (rest) variables in terms of “the average AA property for each protein” [20] in respect of given regression models (Table 1). Such an evaluation revealed that the groups of selected and non-selected AA frequencies can make substantially different contributions to the combined set of average physicochemical [25] and structural [26] properties for the enzyme sequences (Figure 5).

**Figure 6:**
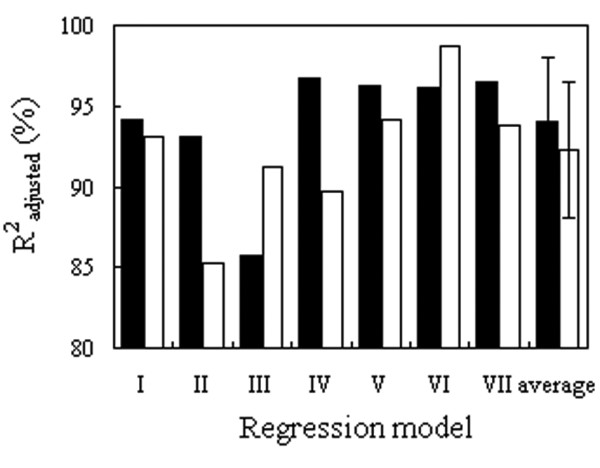
**Adjusted coefficients of determination for the multiple regression models which represent the full AA sequences (filled bars) and those which do not take into account the quantities of catalytic and binding residues in the active sites of enzymes and formed by the recalculated AA frequencies (open bars).** Both model types contain the same independent variables.

Compiling the data [16] on the enzyme active sites, 63 residues representing 11 AA (E, H, K, D, R, N, T, G, S, C, Y) were found to be responsible for the activity of the nine studied enzymes. These almost exclusively charged (E, H, K, D, R) or polar (N,T,S,C,Y) residues represent only a small portion (up to 1.5%) of the total amount (4,406 residues) in the sequences. Even those active site residues also involved as variables (K, D, H, R, N) in the regression models (Table 1) constitute rather low proportion (2.74–3.14%) of their total number in sequences, as well as both sets of frequencies are not correlated. These considerations suggest that the AA represented in the regression models (Table 1) are mainly eligible for the so-called structural residues [3] in enzymes, since the contribution of active center AA frequencies might not be great. This was supported by further control applications of the regression models when the active center AA were “excluded” from the dataset, overall AA frequencies recalculated and the same variables (Table 1) employed. As a result, *R*2 values of the regression models were affected (Figure 6), to a limited extent and close to the proportion of active site residues in sequences whereas all the multiple regressions remained at a high level of statistical significance. Nevertheless, it was observed that the small and unevenly distributed active center frequencies, independently of the overall AAC of the enzyme sequences, can also form multiple linear regressions with the kinetic constants. Thus, the selected sets of relevant variables (E, H, K, S), (N, D, S, T, Y), and (R, H, K, T) form highly significant (*p* < 0.00001) multiple linear regressions with the values of *k*cat, *K*M, and *k*cat/*K*M, respectively, as well as reach the high values of determination coefficients (*R*adj.2: 89.14, 97.63, and 98.84%, respectively). The full set of the respective results is summarized in Additional file 3: Table S3.

It is noted that statistically robust multivariate relationships could also occur in cases where the values of kinetic constants have come from different sources. Thus, the *K*M values which are represented for only seven enzymes of *S. cerevisiae* TCA cycle in the BRENDA database [15] were found to be closely related (*R*adj.2 = 91.81%; *p* = 0.0006) to the selected frequencies of AA (A, R, L, M, P). Besides, the *K*M values included in the Teusink’s model for yeast glycolysis [27] also were closely linked to the frequencies of selected AA (K, Y, C, M, I) in sequences of 10 corresponding enzymes (*R*adj.2 = 98.87%; *p* = 0.0001). Extended sets of these results are summarized in Additional file 4: Figure S1 and Additional file 5: Figure S2, respectively. In this case, the essential differences between the sets of variables for regression models (Table 1) are due to the fact that the *K*M values included in BRENDA have been obtained in “optimized” *in vitro* conditions, while the model uses the estimates (experimental and computational) which are more in line to the environment of living cell [27,28].

## Discussion

The obtained results indicate that the basic kinetic constants [17,18] of yeast glycolytic enzymes appear as closely related to the AAC of the sequences and, therefore, support the view on the actual interdependence of catalytic, binding, and structural residues to ensure the full-scale efficiency of biocatalysts [3] as well as suggest that a certain functional overlap may occur between these sets of AA [6]. Furthermore, the observed relationships fit well with the up-to-date concepts on the structural and functional properties of proteins, including structural, energy and conformational networks [28], conformational dynamics, heterogeneity and selection [7], AA networks [12,29]. A broad representation of AA frequencies as the strong predictor variables for the developed regression models (Table 1) as well as findings about the different impact of the selected AA groups on predicted features of enzyme sequences (Figure 5) most likely reflect the potential of protein adjustments to keep the kinetic parameters of enzymes within a definite range and, consequently, their efficient operation under varied external conditions.

In general, such relationships between the kinetic constants and AAC of the enzymes might include the quadratic effects and interactions between the variables actually making them more complex. Nevertheless, it should be noted that a multiple linear regression still offers a best linear approximation to the unknown regression function even if it is nonlinear [30]. Really, the refinement of the observed multiple linear regressions (Figure 2) by means of the second-order polynomial equations resulted in a marked reduction of unexplained variance which characterize substantially stronger relationships between the variables (Additional file 6: Figure S3). However, it should be taken into account that the practical use of second-order equations are strongly restricted due to a sharp increase of required regression coefficients and degrees of freedom to obtain statistically robust regression models.

It should be noted that this study well corresponds to a certain line of research in recent years where the set of primary structure-derived features [31,32] or integral physico-chemical indices of proteins [33] have been used to predict the values of kinetic constants for particular enzymes.

## Conclusions

The multivariate linear relationships broadly confirm the actual link between the kinetic constants of yeast enzymes and the AAC of the respective sequences. The results of this study suggest to some possible outputs. Regression models of such kind could be used, at least in principle, to specify and co-ordinate the appropriate values of kinetic constants especially if there is a need to include any additional enzyme currently not represented in a given metabolic pathway (e.g., metabolic engineering, dynamic modeling). There is a possibility that the metabolic fluxes could be directly linked to the enzyme sequence-dependent properties including AAC, in particular because they are largely determined by enzyme kinetic parameters [1].

Although, prospects of such an approach apparently now are rather limited due to lack of necessary kinetic parameters and, therefore, are dependent on further data accumulation and specification in the enzyme databases.

## Abbreviations

AA: Amino acid; AAC: Amino acid composition; *k*cat: Turnover number; *K*M: Michaelis-Menten constant; *k*sp: Specificity constant; LOOCV: Leave-one-out cross-validation; VIF: Variance inflation factor.

## Competing interests

The authors declare that they have no competing interests.

## Supplementary Material

Additional file 1 Table S1 Kinetic constants and enzyme AA sequences of the yeast S. cerevisiae. Click here for file

Additional file 2 Table S2 The variance analysis of the regression models. Click here for file

Additional file 3 Table S3 The characteristics of the models obtained by using the set of AA from enzyme active sites.) Click here for file

Additional file 4 Figure S1 The linkage of kinetic constants and AAC for enzymes of the TCA pathway. Click here for file

Additional file 5 Figure S2 The linkage of kinetic constants and AAC for glycolytic enzymes employed in the Teusink’s model. Click here for file

Additional file 6 Figure S3 The second-order multiple relationships of kinetic constants and AAC. Click here for file
