# Supplementary material for: Relationships between kinetic constants and the amino acid composition of enzymes from the yeast Saccharomyces cerevisiae glycolysis pathway
Source: EURASIP J Bioinform Syst Biol. 2012 Aug 6;2012(1):11. doi: 10.1186/1687-4153-2012-11 (PMC3494524; doi:10.1186/1687-4153-2012-11)
Supplement: Additional file 1 — Table S1 Kinetic constants and enzyme AA sequences of the yeast S. cerevisiae. [file 1687-4153-2012-11-S1.pdf]

Additional file 1

Kinetic constants and enzyme amino acid sequences of the yeast *Saccharomyces cerevisiae*

Enzyme (Substrate)	UniProtKB accession number [a]	Kinetic constants [b]		
		k_{cat} s^{-1}	K_M mM	$K_{sp}=k_{cat}/K_M$ $s^{-1}mM^{-1}$
Hexokinase<br>EC 2.7.1.1 HXK (D-glucose)	P04806	1.06	0.120	8.333
Glyceraldehyde-3-phosphate dehydrogenase<br>EC 1.2.1.12 GADH (D-glyceraldehyde 3-phosphate)	P00360	16.70	0.600	27.833
Phosphoglycerate kinase<br>EC 2.7.2.3 PGK (3-phospho-D-glycerate)	P00560	354.00	0.770<br>1.280	459.740<br>276.563
Phosphoglycerate mutase<br>EC 5.4.2.1 PGM (3-phosphoglycerate)	P00950	384.00<br>490.00	0.650<br>0.740	590.769<br>662.162
Enolase<br>EC 4.2.1.11 ENO (2-phospho-D-glycerate)	P00924	78.00<br>230.00	0.043<br>0.050	1813.950<br>4600.000
Pyruvate kinase<br>EC 2.7.1.40 PK (phosphoenolpyruvate)	P00549	58.40<br>66.00<br>226.00<br>232.00	0.021<br>0.045	3142.850<br>5022.220
Pyruvate decarboxylase<br>EC 4.1.1.1 PDC (pyruvate)	P06169	2.10<br>10.00	2.290	0.917<br>1.562
Triosephosphate isomerase<br>EC 5.3.1.1 TIM (D-glyceraldehyde 3-phosphate)	P00942	4700.00<br>6400.00<br>6900.00	1.100<br>1.130<br>1.400	4571.429<br>4928.571<br>5663.717
Pyruvate carboxylase<br>EC 6.4.1.1 PYC (pyruvate)	P11154	60.00	0.495	121.212

a Protein knowledge base UniProtKB/Swiss Prot [15] accession numbers URL: http://www.uniprot.org
b Comprehensive Enzyme Information system BRENDA [14] URL: http://www.brenda-enzymes.org
