# Supplementary material for: Relationships between kinetic constants and the amino acid composition of enzymes from the yeast Saccharomyces cerevisiae glycolysis pathway
Source: EURASIP J Bioinform Syst Biol. 2012 Aug 6;2012(1):11. doi: 10.1186/1687-4153-2012-11 (PMC3494524; doi:10.1186/1687-4153-2012-11)
Supplement: Additional file 2 — Table S2 The variance analysis of the regression models. [file 1687-4153-2012-11-S2.pdf]

Additional file 2

The variance analysis of the regression models. Multiple linear regression models[a] linking the log-values of kinetic constants and the amino acid composition of the yeast *Saccharomyces cerevisiae* enzyme sequences

Model	Variables		Variance source	Sum of squares	Df	Mean square	F-ratio	*p*-value
	dependent	independent						
I	$\log(k_{cat})$	M, W, R, L	model<br>*residual*<br>total	18.3210<br>0.8473<br>19.1683	4<br>12<br>16	4.58024<br>0.07061	64.86	0.0000
II	$\log(k_{cat})$	T, V, H, A, K	model<br>*residual*<br>total	18.2526<br>0.9157<br>19.1683	5<br>11<br>16	3.65052<br>0.08325	43.85	0.0000
III	$\log(k_{cat})$	A, H, V, E	model<br>*residual*<br>total	17.1176<br>2.0507<br>19.1683	4<br>12<br>16	4.27939<br>0.17090	24.50	0.0000
IV	$\log(K_M)$	D, N, W, L, A	model<br>*residual*<br>total	6.1371<br>0.1331<br>6.2702	5<br>9<br>14	1.22741<br>0.01479	82.97	0.0000
V	$\log(k_{cat}/K_M)$	A, H, R, G, Q, N	model<br>*residual*<br>total	21.9479<br>0.4995<br>22.4474	6<br>9<br>15	3.65800<br>0.05550	65.91	0.0000
VI	$\log(k_{cat}/K_M)$	L, T, N, W, Q, F	model<br>*residual*<br>total	21.9286<br>0.5188<br>22.4474	6<br>9<br>15	3.65480<br>0.05764	63.40	0.0000
VII	$\log(k_{cat}/K_M)$	T, Q, C, N, A	model<br>*residual*<br>total	21.7742<br>0.6732<br>22.4474	5<br>10<br>15	4.35480<br>0.06732	64.69	0.0000

a Model elements together with the statistical indices are represented in Table 1
