# Supplementary material for: Relationships between kinetic constants and the amino acid composition of enzymes from the yeast Saccharomyces cerevisiae glycolysis pathway
Source: EURASIP J Bioinform Syst Biol. 2012 Aug 6;2012(1):11. doi: 10.1186/1687-4153-2012-11 (PMC3494524; doi:10.1186/1687-4153-2012-11)
Supplement: Additional file 3 — Table S3 The characteristics of the models obtained by using the set of AA from enzyme active sites.) [file 1687-4153-2012-11-S3.pdf]

Additional file 3

The characteristics of the models obtained by using the set of amino acids from enzyme active sites. Elements and the statistical indices for multiple linear regression models which link the log-values of kinetic constants and the amino acid composition of the yeast *Saccharomyces cerevisiae* enzyme sequences.

Dependent variable	Parame-ters [a]	Regression coefficient	S.E.	*t* value	P value	R^2 %	$R^2_{adjusted}$ %	VIF [b]	R^2 % [c]	$R^2_{adjusted}$ % [c]
$\log(k_{cat})$	*constant*	0.7615	0.2563	2.9714	0.0117	91.86	89.14		84,47	83,44
	E	-2.9006	0.5299	-5.4737	0.0001			2.569		
	H	2.1919	0.4764	4.6008	0.0006			1.895		
	K	7.9553	0.7964	9.9892	0.0000			2.766		
	S	-6.2860	1.4845	-4.2345	0.0012			2.422		
$\log(K_M)$	*constant*	-0.1832	0.0565	-3.2398	0.0098	98.48	97.63		89,72	88,93
	N	0.6671	0.2046	3.2609	0.0000			1.321		
	D	-1.6721	0.1319	-12.6784	0.0000			1.865		
	S	-4.1560	0.4235	-9.8128	0.0094			1.659		
	T	0.9062	0.2757	3.2872	0.0001			2.324		
	Y	4.0621	0.6085	6.6759				1.029		
$\log(k_{cat}/K_M)$	*constant*	-0.5246	0.0975	-5.3805	0.0002	99.15	98.84		98,15	98,02
	R	1.8406	0.1482	12.4207	0.0000			1.117		
	H	3.1678	0.1538	20.5944	0.0000			1.283		
	K	7.2704	0.2140	33.9712	0.0000			1.264		
	T	2.0057	0.2548	7.8726	0.0000			1.137		

a Elements of multiple linear regression which represent the frequences of amino acid (a single letter code) from occurrence in the yeast *Saccharomyces cerevisiae* enzyme active sites and the constant (intercept) of equation

b The variance inflation factor which indicates the impact of multicollinearity between the independent variables [22]

c Obtained by the leave-one-out cross-validation (LOOCV) [21] of models
