# Supplementary material for: Relationships between kinetic constants and the amino acid composition of enzymes from the yeast Saccharomyces cerevisiae glycolysis pathway
Source: EURASIP J Bioinform Syst Biol. 2012 Aug 6;2012(1):11. doi: 10.1186/1687-4153-2012-11 (PMC3494524; doi:10.1186/1687-4153-2012-11)

Additional file 5

The linkage of kinetic constants and amino acid composition for glycolytic enzymes employed in the Teusink's model.

Relationship between the amino acid composition and Km values for 10 enzymes (HXK, GPI, ALD, GAPDH, GlycPDH, PGK, PGM, ENOL, PK, PDC) employed in the Teusink′s model [26] for yeast glycolysis. The plot of actual Km values (A) versus those predicted by the linear regression equation: $\log(K_M)=5.1877 -0.84811*K -0.73666*C +0.46523*Y -0.40875*M +0.13872*P$ ($R^2_{adj.}=98.87\%$, $p=0.0000$). Relationship between an increase in the percentage of explained variance and the number of independent variables (amino acid frequencies of occurrence) included in multiple regressions (B), where: 1 – K, 2 – K, Y, 3 – K, Y, C, 4 – K, Y, C, M, and 5 – K, Y, C, M, I.

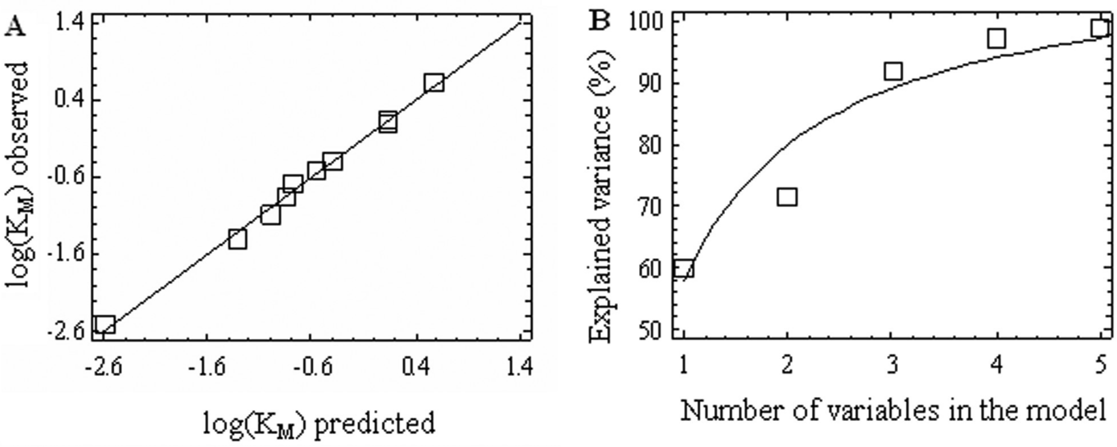

Supplement: Additional file 5 — Figure S2 The linkage of kinetic constants and AAC for glycolytic enzymes employed in the Teusink’s model. [file 1687-4153-2012-11-S5.pdf]
