# Supplementary material for: Relationships between kinetic constants and the amino acid composition of enzymes from the yeast Saccharomyces cerevisiae glycolysis pathway
Source: EURASIP J Bioinform Syst Biol. 2012 Aug 6;2012(1):11. doi: 10.1186/1687-4153-2012-11 (PMC3494524; doi:10.1186/1687-4153-2012-11)

Additional file 6

The 2nd order multiple relationships of kinetic constants and amino acid composition. The 2nd order multiple regressions showing changes of the log-values of kinetic constants as dependent variables upon the frequences of occurrence for two amino acids in the yeast *Saccharomyces cerevisiae* enzyme sequences, where k_{cat} is the catalytic constant (A), K_M is the Michaelis-Menten constant (C), and k_{cat}/K_M is the specificity constant k_{sp} (E). The observed versus predicted plots (B,D,F) for the values of dependent variables (k_{cat}, K_M, and k_{cat}/K_M, respectively). The predicted values were calculated from the regression equations: $\log(k_{cat})$= 2.292 +2.644*W -0.584*W^2 -1.634*M*W ($R^2_{adj.}$=92.36%, p=0.0000); $\log(K_M)$= -15.938 +10.145*N -1.035*N^2 -0.311*D*N ($R^2_{adj.}$= 79.40%, p=0.0001); $\log(k_{sp})$=5.847 -4.251*H +0.247*A*H ($R^2_{adj.}$=51.71%, p=0.0035).

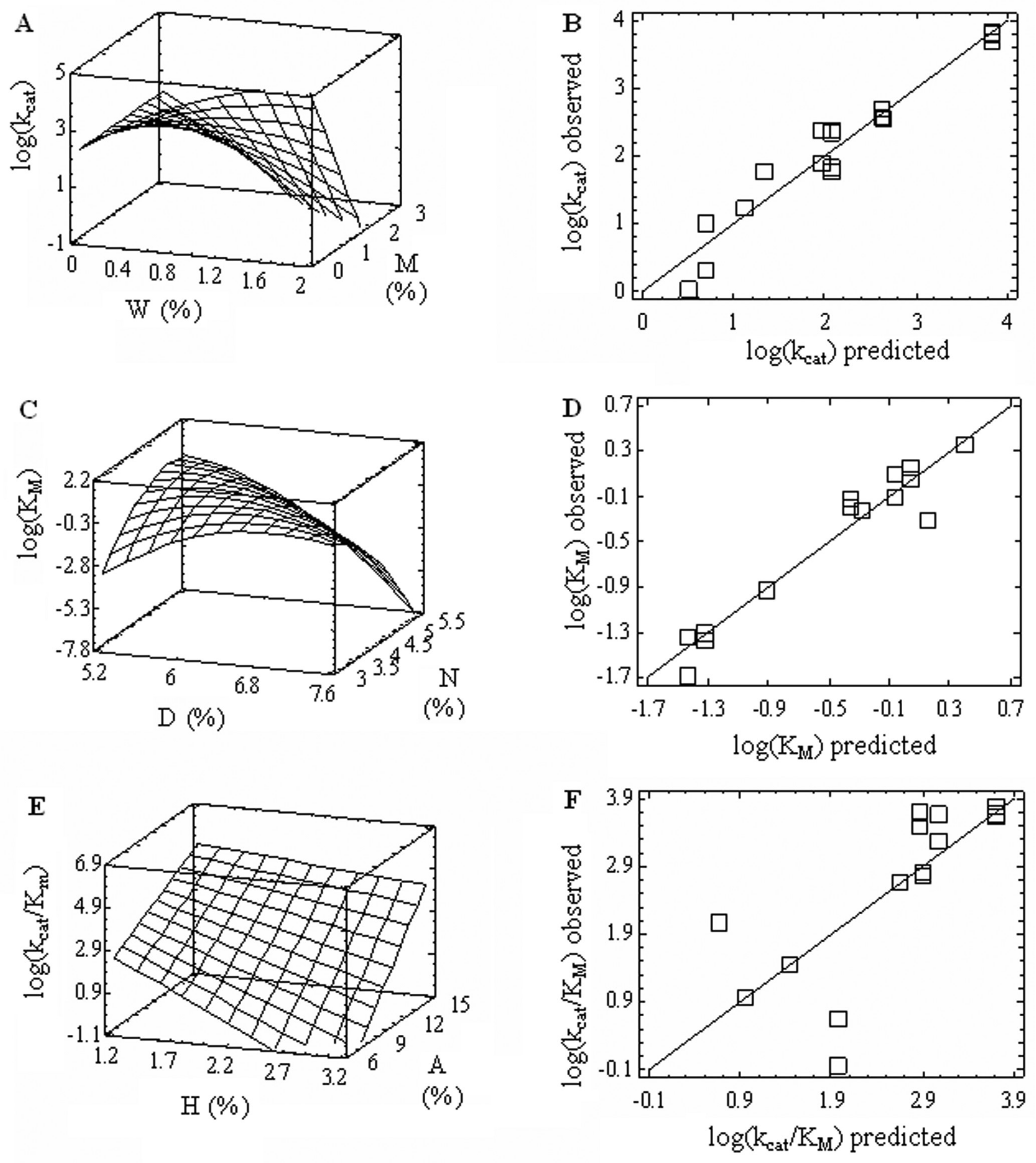

Supplement: Additional file 6 — Figure S3 The second-order multiple relationships of kinetic constants and AAC. [file 1687-4153-2012-11-S6.pdf]
